# A nomogram for predicting breast cancer specific survival in elderly patients with breast cancer: a SEER population-based analysis

**DOI:** 10.1186/s12877-023-04280-8

**Published:** 2023-09-25

**Authors:** Ruoning Yang, Yunhao Wu, Yana Qi, Weijing Liu, Ya Huang, Xin Zhao, Ruixian Chen, Tao He, Xiaorong Zhong, Qintong Li, Li Zhou, Jie Chen

**Affiliations:** 1 Division of Breast Surgery, Department of General Surgery, West China Hospital, Sichuan University, Chengdu, China; 2 Breast Center, West China Hospital, Sichuan University, Chengdu, China; 3 Chinese Evidence-Based Medicine Center, West China Hospital, Sichuan University, Chengdu, China; 4 Departments of Obstetrics & Gynecology and Pediatrics, West China Second University Hospital, Key Laboratory of Birth Defects and Related Diseases of Women and Children, Ministry of Education, Development and Related Diseases of Women and Children Key Laboratory of Sichuan Province, Center of Growth, Metabolism and Aging, Sichuan University, Chengdu, Sichuan 610041 China; 5 Departments of Obstetrics & Gynecology and Pediatrics, West China Second University Hospital, Key Laboratory of Birth Defects and Related Diseases of Women and Children, Ministry of Education, State Key Laboratory of Biotherapy and Collaborative Innovation Center of Biotherapy, Sichuan University, Chengdu, 610041 China; 6 Public Experimental Technology Center, West China Hospital, Sichuan University, Chengdu, China; 7 Department of Breast Surgery, West China Hospital, Sichuan University, Guoxue Street 37#, Chengdu, 610041 China

**Keywords:** Breast cancer, Elderly patients, Nomogram, Overall survival, Prognostic model, Risk stratification

## Abstract

**Background:**

The number of elderly patients diagnosed with breast cancer is increasing worldwide. However, treatment decisions for these patients are highly variable. Although researchers have identified the effects of surgery, radiotherapy, endocrine therapy, and chemotherapy in elderly patients with breast cancer, clinicians still struggle to make appropriate decisions for these patients.

**Methods:**

We identified 75,525 female breast cancer patients aged ≥ 70 years in the Surveillance, Epidemiology, and End Results (SEER) database treated between January 1, 2010, and December 31, 2016. The patients were further divided into training and testing cohorts. The cumulative occurrence of breast cancer-specific deaths (BCSDs) and other cause-specific deaths (OCSD) was calculated using the cumulative incidence function. In the univariate analysis, risk factors were screened using the Fine-Gray model. In the multivariate analysis for competing risks, the sub-distribution hazard ratio with a 95% confidence interval for each independent predictor associated with BCSD was calculated for the construction of nomograms. Based on the above analyses, a competing risk nomogram was constructed to predict the probability of BCSD in the 1st, 3rd, and 5th years after treatment. During validation, the concordance index (C-index) was selected to quantify the predictive ability of the competing risk model.

**Results:**

A total of 33,118 patients were included in this study, with 24,838 in the training group and 8,280 in the testing group. Age, race, marital status, cancer grade, tumor stage, node stage, estrogen receptor status, progesterone receptor status, human epidermal growth factor receptor--2 status, and treatment including surgery, radiation, and chemotherapy were used to establish a nomogram. The C-index of 0.852 (0.842-0.862) in the training cohort and 0.876 (0.868-0.892) in the testing cohort indicated satisfactory discriminative ability of the nomogram. Calibration plots showed favorable consistency between the nomogram predictions and actual observations in both the training and validation cohorts.

**Conclusions:**

Our study identified independent predictors of BCSD in elderly patients with breast cancer. A prognostic nomogram was developed and validated to aid clinical decision-making.

**Supplementary Information:**

The online version contains supplementary material available at 10.1186/s12877-023-04280-8.

## Background

Breast cancer is the most common cancer and cause of cancer-related death in women, and its incidence is positively correlated with age [1, 2]. Approximately 50% of new breast cancer cases are recorded in women ≥ 60 years (https://gco.iarc.fr/). However, treatment decisions for elderly patients with breast cancer are highly variable [3, 4]. On the one hand, aging is accompanied by fragility and comorbidities [5, 6]. On the other hand, prospective studies supporting specific treatments for elderly patients with breast cancer are lacking owing to ethical requirements [7]. Therefore, no uniform treatment guidelines have been established for the elderly [8, 9].

Some studies have been conducted in elderly patients with breast cancer whose primary option is surgery [10]. The choice of surgical method is affected by age; that is, the acceptance rate of breast-conserving surgery decreases with age, and some studies have focused on the unwillingness of patients to receive the necessary radiotherapy after breast-conserving surgery [11, 12]. Elderly patients exhibit a negative attitude toward their choice of treatment strategy, with a low reception of radiotherapy and chemotherapy. Among patients with indications for radiotherapy, only two thirds patients aged 71–80 years received this treatment [13]. Many older patients receive inadequate chemotherapy treatment [14]. A study showed that the proportion of patients aged ≥ 65 years with breast cancer who received a sufficient number of chemotherapy courses was significantly lower than that in younger patients (*P* < 0.001 [15]. Despite an understanding of breast cancer treatment in elderly individuals, clinicians still struggle to make appropriate treatment decisions for these individuals. To solve this problem, we analyzed the competing risks of breast cancer patients over 70 years of age using the Surveillance, Epidemiology, and End Results (SEER) database, identified independent predictors of breast cancer-specific death (BCSD), and constructed a nomogram of a predictive risk model to aid in clinical decision-making.

## Methods

### Data sources and patient selection

This study was based on SEER database data released in November 2020. Our target patients were extracted from SEER*Stat Version 8.3.9.2 (SEER ID: 26588-Nov2019), which included population-based data from 18 cancer registries covering approximately 28% of the United States (U.S.) cancer population between 1975 and 2018 and provided complete data regarding patient demographics, tumor characteristics, diagnosis, first course of treatment, and follow-up of vital status. Given that the data released by the SEER database are publicly available, this study did not require informed patient consent or ethical approval. We extracted data on patients with breast cancer, including chemotherapy records, who were treated between January 1, 2010, and December 31, 2016. A total of 252,472 breast cancer cases were identified in the database during this period (Supplementary 1). Among them, patients who met any of the following criteria were excluded:1) male sex; 2) aged <70 years at diagnosis; 3) breast cancer was not the first primary cancer diagnosed; 4) paired site; 5) without histologic confirmation; 6) missing stage or stage 0; 7) missing molecular type; 8) missing grade; 9) distant metastasis; or 10) death or loss to follow-up within six months of diagnosis. Ultimately, 33,118 eligible patients were included in the analysis. These patients were randomized at a 2:1 ratio into the training and testing groups.

### Data acquisition

We collected patient data, including age at diagnosis, race (white, Black, other, or unknown), marital status (married, divorced, separated, single, widowed, unmarried, domestic partner, or unknown), insurance status (insured, insured/no specifics, any medical, uninsured, or insurance unknown), grade (G1, G2, or G3), stage (I, II, III, or IV), tumor/node/metastasis (TNM) stage (T0–T4, N0–N3, or M0–M1), estrogen receptor (ER) status (negative, positive, or borderline), progesterone receptor (PR) status (negative, positive, or borderline), human epidermal growth factor receptor02 (HER2) status (negative, positive, or borderline), breast surgery procedure (partial mastectomy with or without axillary dissection, simple and subcutaneous mastectomy, modified radical mastectomy, radical and extended radical mastectomy with or without breast reconstruction, and other mastectomy or unknown), and chemotherapy and radiotherapy records. We defined the TNM stage according to the 7th edition guidelines of the American Joint Committee on Cancer (2010–2015). Detailed information about the variables can be found on the official SEER website (https://seer.cancer.gov/data-software/documentation/seerstat/nov2020/), and we strictly followed these definitions while conducting the analysis.

### Outcomes

We defined BCSD as the time from diagnosis to death due to breast cancer. Death from other causes was defined as other cause-specific death (OCSD). We used the description from “SEER cause-specific death classification” to define the patient's cause of death.

### Statistical analysis

We used a chi-square test to compare categorical variables. The cumulative occurrence of BCSD and OCSD was calculated using the cumulative incidence function (CIF). The difference between BCSD and OCSD CIFs in different subgroups, including those defined by age, race, insurance, marital status, grade, T stage, N stage, ER status, PR status, HER2 status, surgery method, and treatment with radiation or chemotherapy, were first compared using Gary’s test. Subsequently, the 1-, 3-, and 5-year CIFs for BCSD and OCSD in patients with breast cancer in the training cohort were calculated. In univariate analyses, risk factors were screened using the Fine-Gray model, and values with *P* < 0.05 were included in the subsequent multifactor analysis [16]. In the multivariate analysis for competing risks, the sub-distribution hazard ratio (sdHR) with a 95% confidence interval (CI) of each independent predictor associated with BCSD was calculated for the construction of nomograms. Based on the above analyses, a competing risk nomogram was constructed to predict the probability of BCSD in the 1st, 3rd, and 5th years after treatment [17]. The nomograph was verified using the 1000 resampling guidance method to assess its performance internally and externally. The concordance index (C-index) was chosen to quantify the predictive ability of the competing risk model [18]. The C-index ranges from 0.5 to 1, with values greater than 0.7 indicating better discrimination performance. And calibration curves were used to compare the predicted probability and observed frequencies, and the location of curve is closer to a 45° diagonal line, meaning a better-calibration. All analyses were performed using R software (version 4.1.3), and all tests were two-sided, and statistical significance was set at *P* < 0.05.

## Results

### Clinicopathological and baseline characteristics of patients

A total of 33,118 patients were included in this study, with 24,838 in the training group and 8,280 in the testing group. Clinicopathological and baseline characteristics are presented in Table 1.

**Table 1:** Clinicopathologic and baseline characteristics of patients

	**All patients**		**Training cohort**		**Testing cohort**	
	*N* = 33,118		*N* = 24,838		*N* = 8280	
**Variable**	No	%	No	%	No	%
**Age**						
** 70–80**	21,954	66.3	16,494	66.4	5460	65.9
** 80–90**	9710	29.3	7289	29.3	2421	29.2
** 90 + **	1454	4.4	1055	4.2	399	4.8
**Race**						
**White**	27,296	82.4	20,483	82.5	6813	82.3
**Black**	2533	7.6	1866	7.5	667	8.1
**Others**	3289	9.9	2489	10.0	800	9.7
**Insurance**						
**No**	91	0.3	71	0.3	20	0.2
**Yes**	32,543	98.3	24,412	98.3	8131	98.2
**Unknow**	484	1.5	355	1.4	129	1.6
**Marital status**						
**No**	17,335	52.3	12,971	52.2	4364	52.7
**Yes**	14,176	42.8	10,669	43.0	3507	42.4
**Unknow**	1607	4.9	1198	4.8	409	4.9
**Grade**						
**1**	9606	29.0	7262	29.2	2344	28.3
**2**	15,537	46.9	11,587	46.7	3950	47.7
**3**	7975	24.1	5989	24.1	1986	24.0
**T**						
**0**	14	0.0	12	0.0	2	0.0
**1**	21,106	63.7	15,862	63.9	5244	63.3
**2**	9610	29.0	7174	28.9	2436	29.4
**3**	1537	4.6	1150	4.6	387	4.7
**4**	851	2.6	640	2.6	211	2.5
**N**						
**0**	25,493	77.0	19,097	76.9	6396	77.2
**1**	5650	17.1	4245	17.1	1405	17.0
**2**	1246	3.8	950	3.8	296	3.6
**3**	729	2.2	546	2.2	183	2.2
**ER**						
**Negative**	4130	12.5	3077	12.4	1053	12.7
**Positive**	28,988	87.5	21,761	87.6	7227	87.3
**PR**						
**Negative**	7912	23.9	5882	23.7	2030	24.5
**Positive**	25,206	76.1	18,956	76.3	6250	75.5
**HER2**						
**Negative**	29,822	90.0	22,386	90.1	7436	89.8
**Positive**	3296	10.0	2452	9.9	844	10.2
**Surgery**						
**No**	1558	4.7	1179	4.7	379	4.6
**Partial mastectomy**	27,429	82.8	20,564	82.8	6865	82.9
**Mastectomy**	4080	12.3	3055	12.3	1025	12.4
**Other or unknown**	51	0.2	40	0.2	11	0.1
**Radiation**						
**No**	17,260	52.1	12,933	52.1	4327	52.3
**Yes**	15,858	47.9	11,905	47.9	3953	47.7
**Chemotherapy**						
**No**	27,859	84.1	20,878	84.1	6981	84.3
**Yes**	5259	15.9	3960	15.9	1299	15.7

*T* tumor stage, *N* nearby lymph node stage, *ER* estrogen receptor, *PR* progesterone receptor, *HER2* growth factor receptor 2

Among all patients, 21,954 (66.3%) were aged between 70 and 80 years, 9,710 (29.3%) were aged between 80 and 90 years, and 1,454 (4.4%) were older than 90 years. Among all patients, 12.5% were ER-negative, 23.9% were PR-negative, and 90% were HER2-negative. In the entire population, a vast majority of patients underwent surgery, 82.8% of whom underwent partial mastectomy and 12.3% underwent mastectomy. Approximately half of the patients were treated with radiation; however, only a small proportion (15.9%) received chemotherapy.

### Univariate and multivariate analyses

**Fig. 1:**
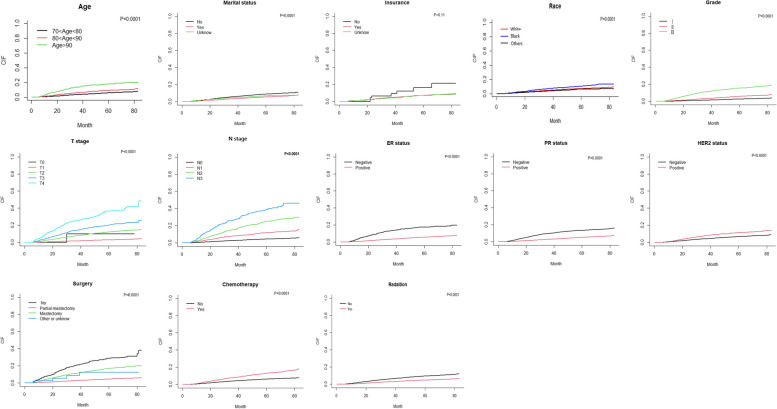
The univariate analysis with the CIF of BCSD by Grays-test

In the training cohort, the number of patients (12.4%; 3,083/24,838) who died from non-breast cancer-related causes was higher than that of patients (5.7%; 1,412/24,838) who died from breast cancer. The 1-, 3-, and 5-year CIFs of BCSD and OCSD in patients with breast cancer in the training cohort are shown in Table 2 and Supplementary Table 2, respectively. The 1-, 3-, and 5-year CIFs of BCSD among the patients were 0.94%, 4.51%, and 7.23%, respectively, and those of OCSD were 1.46%, 8.11%, and 16.06%, respectively, which were almost twice the CIFs of BCSD. In the univariate analyses, most variables (*P* < 0.05) strongly correlated with the CIF of the BCSD, except for insurance (*P* = 0.110). Differences in the CIF of the BCSD are shown in Fig. 1. There was no relationship between CIF and ER status (*P* = 0.680), PR status (*P* = 0.069), HER2 status (*P* = 0.771), or N stage (*P* = 0.919). Multivariate analysis revealed the independent risk factors (age, race, marital status, grade, T stage, N stage, ER status, PR status, HER2 status, and treatment with surgery, radiation, or chemotherapy) associated with BCSD for the subsequent construction of a nomogram. The results of the multivariate analysis of competing risks in the training group are presented in Table 3. Among the identified factors, age, grade, T stage, and N stage positively correlated with the CIF of BCSD. Black and unmarried women have a higher risk of developing BCSD than those of other races and martial statuses. According to the sdHRs with 95% CI, the possibility of BCSD increased with grade, as observed in the T and N stages. Compared to patients who did not receive radiation or chemotherapy, those who underwent radiation (sdHR 0.760 [0.661–0.873]) or chemotherapy (sdHR 0.580 [0.514–0.653]) had a reduced probability of BCSD.

**Table 2:** 1-, 3-, 5-Year CIF of BCSD among patients with breast cancer in the training cohort

**Variable**	**N**	**1-y (%)**	**3-y (%)**	**5-y (%)**	***p***
**Total**	1412	0.94	4.51	7.23	
**Age**					< 0.0001
**70–80**	719	0.59	3.31	5.71	
**80–90**	583	1.28	5.90	9.06	
**90 + **	155	4.09	13.53	17.74	
**Race**					< 0.0001
** White**	1153	0.92	4.40	7.07	
** Black**	151	1.46	7.01	10.31	
** Others**	108	0.69	3.60	6.35	
**Marital status**					< 0.0001
** No**	898	1.18	5.57	8.78	
** Yes**	449	0.59	3.19	5.40	
** Unknow**	65	1.43	4.84	6.56	
**Grade**					< 0.0001
** 1**	141	0.12	1.30	2.64	
** 2**	504	0.57	3.05	5.77	
** 3**	767	2.64	11.16	15.44	
**T**					
** 0**	1	0.00	10.00	10.00	< 0.0001
** 1**	364	0.29	1.64	2.94	
** 2**	678	1.54	7.54	12.12	
** 3**	184	3.24	13.93	20.36	
** 4**	185	6.29	25.25	36.55	
**N**					< 0.0001
** 0**	642	0.48	2.58	4.33	
** 1**	393	1.85	7.88	11.79	
** 2**	193	2.95	15.12	25.01	
** 3**	184	6.45	26.56	38.40	
**ER**					< 0.0001
** Negative**	449	3.62	13.23	17.44	
** Positive**	963	0.56	3.27	5.76	
**PR**					< 0.0001
** Negative**	656	2.32	9.74	13.50	
** Positive**	756	0.51	2.88	5.25	
**HER2**					< 0.0001
** Negative**	1195	0.90	4.16	6.79	
** Positive**	217	1.27	7.79	11.30	
**Surgery**					< 0.0001
** No**	246	5.98	20.47	28.76	
** Partial mastectomy**	718	0.44	2.63	4.56	
** Mastectomy**	444	2.37	11.07	16.86	
** Other or unknown**	4	2.56	8.71	12.37	
**Radiation**					< 0.0001
** No**	969	1.38	6.10	9.47	
** Yes**	443	0.46	2.80	4.79	
**Chemotherapy**					< 0.0001
** No**	1012	0.82	3.88	6.17	
** Yes**	400	1.55	7.90	12.92	

*CIF* Cumulative Incidences Function, *BCSD* Breast Cancer-Specific Death, *T* tumor stage, *N* nearby lymph node stage, *ER* estrogen receptor, *PR* progesterone receptor, *HER2* growth factor receptor 2

**Table 3:** Hazard ratio for BCSD in the training cohort

**Characteristics**	**Coefficient**	**sdHR (95% CI)**	***P*-value**
**Age**			
**70–80**	Reference		
**80–90**	0.424	1.528 (1.355–1.723)	< 0.0001
**90 + **	1.241	3.458 (2.861–4.180)	< 0.0001
**Race**			
**White**	Reference		
**Black**	0.049	1.051 (0.884–1.249)	0.5761
**Others**	-0.410	0.663 (0.544–0.809)	< 0.0001
**Marital status**			
**No**	Reference		
**Yes**	-0.175	0.839 (0.745–0.946)	0.0040
**Unknow**	-0.126	0.881 (0.684–1.135)	0.3269
**Grade**			
**1**	Reference		
**2**	0.371	1.450 (1.198–1.754)	0.0001
**3**	1.058	2.881 (2.365–3.511)	< 0.0001
**T**			
**0**	Reference		
**1**	1.048	2.851 (0.398–20.445)	0.2973
**2**	1.880	6.556 (0.917–46.864)	0.0610
**3**	2.125	8.370 (1.167–60.002)	0.0345
**4**	2.445	11.525 (1.610–82.489)	0.0149
**N**			
**0**	Reference		
**1**	0.555	1.743 (1.520–1.999)	< 0.0001
**2**	1.176	3.240 (2.693–3.898)	< 0.0001
**3**	1.706	5.504 (4.530 -6.688)	< 0.0001
**ER**			
**Negative**	Reference		
**Positive**	-0.465	0.628 (0.534–0.738)	< 0.0001
**PR**			
**Negative**	Reference		
**Positive**	-0.399	0.671 (0.579–0.777)	< 0.0001
**HER2**			
**Negative**	Reference		
**Positive**	-0.204	0.815 (0.699–0.951)	0.0091
**Surgery**			
**No**	Reference		
**Partial mastectomy**	1.371	0.254 (0.215–0.299)	< 0.0001
**Mastectomy**	-1.053	0.349 (0.294–0.414)	< 0.0001
**Other or unknown**	-0.765	0.465 (0.173–1.254)	0.1303
**Radiation**			
**No**	Reference		
**Yes**	-0.546	0.760 (0.661–0.873)	0.0001
**Chemotherapy**			
No	Reference		
**Yes**	-0.275	0.580 (0.514–0.653)	< 0.0001

*BCSD* breast cancer-specific death, *T* tumor stage, *N* nearby lymph node stage, *ER* estrogen receptor, *PR* progesterone receptor, *HER2* growth factor receptor 2

### Construction and validation of the competing risk nomogram

**Fig. 2:**
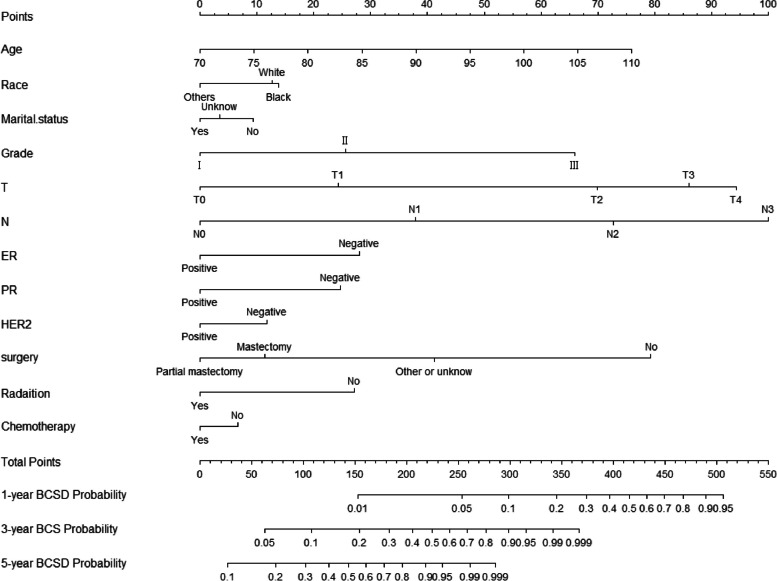
Nomogram to predict the 1-, 3-, and 5-year CIF of BCSD

After model validation, all independent risk factors, including age, race, marital status, grade, T stage, N stage, ER status, PR status, HER2 status, and treatment with surgery, radiation, or chemotherapy were incorporated to construct a nomogram to predict the 1-, 3-, and 5-year CIFs of BCSD, as shown in Fig. 2. The probabilities of BCSD at 1-, 3-, and 5-years were predicted using the total score in the nomogram. As shown in Fig. 2, the N stage had the strongest effect on BCSD, followed by the T stage, age, and breast surgery method.

**Fig. 3:**
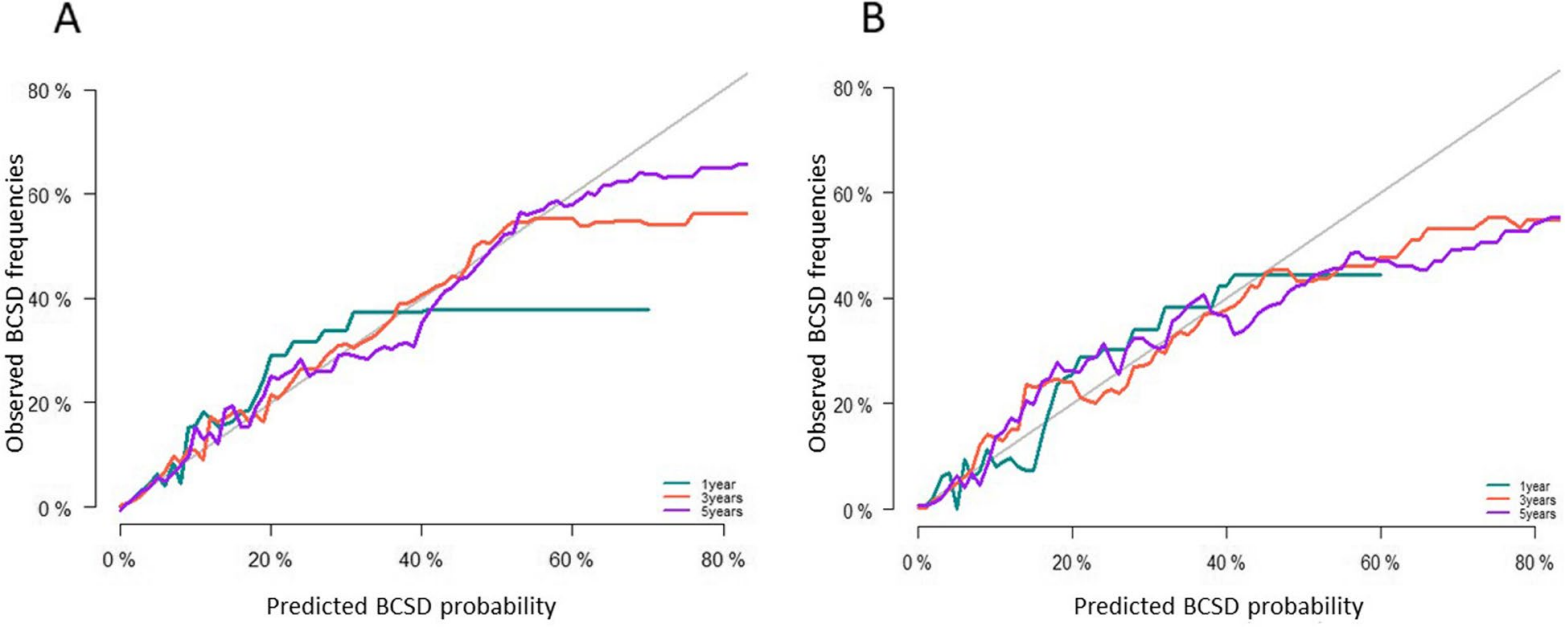
The calibration curves of nomogram-predicted probability of 1-, 3-, and 5-year. **A** Internal calibration curves for 1-, 3-, and 5-year BCSD. **B** External calibration curves for 1-, 3-, and 5-year BCSD

In the internal and external validation, the nomogram showed great predictive ability, with a C-index of 0.852 (0.842-0.862) in the training cohort and 0.876 (0.868-0.892) in the testing cohort, which indicated satisfactory discriminative ability of the nomogram. Calibration plots showed favorable consistency between the nomogram predictions and actual observations in both the training and validation cohorts. The calibration results are shown in Fig. 3.

## Discussion

Breast cancer is the most common malignant tumor and the main cause of death in elderly women [19]. Despite comprising a large proportion of breast cancer cases, the elderly are underrepresented in clinical trials [20], which is related to frailty in the elderly. Understanding the risk factors for BCSD in elderly patients with breast cancer could help comprehensively evaluate the status of patients and is of great importance for treatment decision-making.

The choice of treatment for elderly cancer patients is often complicated by the presence of multiple chronic comorbidities. When discussing the impact of breast cancer on the survival of older patients, deaths from other causes may occur before the event of interest, leading to the exclusion of relevant events. Considering this, a competing risk model was selected to address competing risk events.

In this study, we extracted information on 33,118 elderly patients with breast cancer from the SEER database and constructed a competitive risk model to screen 12 independent risk factors related to BCSD, making the results highly reliable. The probability of BCSD is correlated with age and tumor characteristics, such as molecular classification, tumor grade, and tumor stage. Compared to previous articles that included ten risk factors [21], more risk factors were included in study that impact treatment choices, including radiation and chemotherapy, and BCSD in elderly patients. Elderly patients are likely to choose to forego chemotherapy and radiation because of the higher likelihood of adverse effects. We considered these two treatment approaches; thus, this nomogram can be an effective tool for predicting the CIF of patients with BCSD and appropriate treatment strategies.

In our study, we found that older age was an independent risk factor for higher BCSD probabilities. The inclusion of age as an independent predictive factor for the prognosis of patients with breast cancer has been a subject of ongoing controversy [22]. For patients with breast cancer, especially those younger than 35 years old, younger age is associated with poor prognosis [23, 24]. For elderly breast cancer patients, it is generally observed that the prognosis tends to worsen with increasing age, which is consistent with our results [21, 25]. Regarding tumor factors, tumor stage and grade were important predictive risk factors, having a positive correlation with the CIF of BCSD, consistent with previously reported results [26, 27].

Among these results, the effect of treatment on BCSD in elderly patients was our main focus. Among all treatment methods, surgery showed the greatest impact on BCSD in elderly patients, and this finding is similar to previous studies [28–30]. One study showed that in early stage breast cancer, surgical treatment led to similar 5-year survival rates in both elderly and young patients [28]. Some reports have also shown that age, comorbidities, cognition, functional status, and tumor size are correlated with the preference for operative treatment [31]. With increasing age, few patients are recommended breast-conserving surgery, possibly because of clinicians’ concern that elderly patients cannot tolerate radiotherapy [11, 32, 33]. However, our results suggest that patients who underwent mastectomy had a higher incidence of BCSD than those who underwent breast-conserving surgery.

In our analysis, chemotherapy significantly reduced the incidence of BCSD in elderly patients with breast cancer. Previous studies have reported that the toxicity and side effects of chemotherapy are severe, and the life expectancy of the elderly is short; therefore, the elderly are considered to benefit minimally from chemotherapy [34]. In our study, only 15.4% of patients received chemotherapy, which is an extremely small proportion. Chemotherapy significantly reduces disease-free survival and prolongs overall survival in patients aged < 70 years old [35]. In recent studies, chemotherapy was found to prolong disease-free survival and reduce the relative risk of recurrence among patients with breast cancer aged ≥ 65 years [36]. In addition, chemotherapy has no significant effect on the cognitive function or quality of life in elderly patients receiving this treatment [37, 38]. Therefore, chemotherapy is safe and suitable for elderly patients with breast cancer and has a negligible effect on their quality of life.

Our results show that radiotherapy is more effective than chemotherapy [39, 40]. In early-stage, ER-positive patients aged > 70 years, adjuvant radiotherapy combined with endocrine therapy after breast-conserving surgery or mastectomy can significantly reduce the incidence of local recurrence but has no effect on overall survival [41, 42]. In contrast, ER-negative patients with early-stage breast cancer have better overall survival when treated with radiotherapy [43]. However, due to limited information in the database, regional radiotherapy and postoperative whole-breast radiotherapy cannot be distinguished; therefore, the impact of different radiotherapy modalities on outcomes could not be further analyzed when analyzing the effect of radiotherapy on BCSD. In general, radiotherapy may be recommended for disease control in elderly patients with a life expectancy of 5–10 years, radiotherapy might be recommended to control the disease [44].

Using the SEER database, we constructed a nomogram to predict the CIF of BCSD in elderly patients in the 1st, 3rd, and 5th years after diagnosis. Compared with previous nomograms, our nomogram only focused on elderly patients and included additional clinical risk factors, particularly treatment modalities. Data on clinical factors can be collected from the medical histories at any time. The prediction accuracy of our nomogram was confirmed using the C-index and calibration curves, and the results proved that our nomogram is convenient and reliable. The use of a high-quality and large-sample database to conduct competitive risk analysis makes our study highly reliable.

In the future, clinicians may use this tool to accurately assess the prognosis of elderly patients with breast cancer and provide them with targeted and individualized treatments. Through this nomogram, patients can intuitively understand the benefits of different treatment methods and their prognoses. For example, based on our nomogram, the 1-, 3-, and 5-year BCSD of a 87-year-old patient, who is unmarried, white and with grade III triple-negative breast cancer staged T2 and N0, with partial mastectomy, was 3.84%, 20.5% and 34.7%, respectively.

However, this study has some limitations. Although an extremely small fraction, some cases with missing information were excluded from our analysis, possibly causing selection bias. In addition, our analysis was based on reported data, which may contain information bias. Finally, systemic treatments are being developed, and an increasing number of targeted drugs are being administered in clinics, both of which have a great impact on patient recovery. Although studies have shown that the use of endocrine therapy in elderly patients with breast cancer has become common practice [45], our present work lacks data on endocrine therapy in these patients. Regardless, the lack of data on endocrine therapy did not affect the judgment of the overall results. Finally, the effect of comorbidities on prognosis was not considered in this study. To externally validate our nomogram, a large amount of data from prospective cohort studies is needed.

## Conclusions

Our study identified independent predictors of BCSD in elderly patients with breast cancer and developed and validated a prognostic nomogram to aid clinical decision-making.

### Supplementary Information

**Additional file 1: Supplementary 1.** Patients selection.

**Additional file 2: Supplementary 2.** 1-, 3-, 5-Year CIF of OCSD among patients with breast cancer in the training cohort.

## Data Availability

Since data released by the SEER database (https://gco.iarc.fr/) was publicly available, ethics approval and informed patient consent was not required for this study.

## Abbreviations

BCSD: Breast cancer-specific death
C-index: Concordance index
CI: Confidence interval
CIF: Cumulative incidence function
ER: Estrogen receptor
HER2: Human epidermal growth factor-2
HR: Hazard ratio
PR: Progesterone receptor
SEER: Surveillance, Epidemiology, and End Results
