# Supplementary material for: A nomogram for predicting breast cancer specific survival in elderly patients with breast cancer: a SEER population-based analysis
Source: BMC Geriatr. 2023 Sep 25;23:594. doi: 10.1186/s12877-023-04280-8 (PMC10518930; doi:10.1186/s12877-023-04280-8)
Supplement: Supplementary file 1 — Additional file 1: Supplementary 1. Patients selection. [file 12877_2023_4280_MOESM1_ESM.pdf]

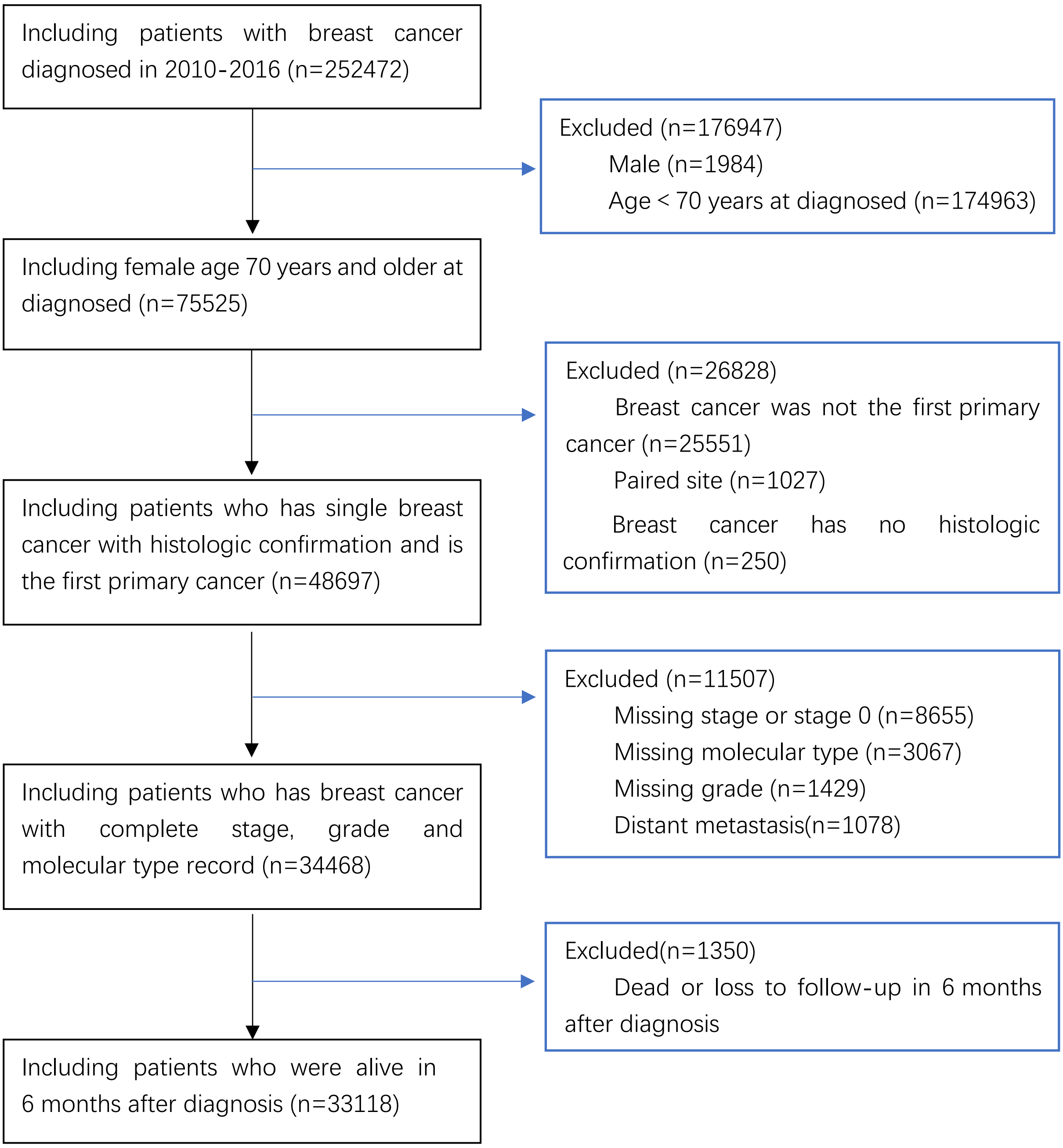

Supplementary 1 Patients Selection

Patients with breast cancer data including treatment record were included from January 1, 2010 to December 31, 2016. 252,472 patients diagnosed breast cancer in the database during this time period were identified . Finally, 33,188 eligible patients were included.
