# Supplementary material for: A nomogram for predicting breast cancer specific survival in elderly patients with breast cancer: a SEER population-based analysis
Source: BMC Geriatr. 2023 Sep 25;23:594. doi: 10.1186/s12877-023-04280-8 (PMC10518930; doi:10.1186/s12877-023-04280-8)
Supplement: Supplementary file 2 — Additional file 2: Supplementary 2. 1-, 3-, 5-Year CIF of OCSD among patients with breast cancer in the training cohort. [file 12877_2023_4280_MOESM2_ESM.doc]

**Supplementary 2 1-, 3-, 5-Year CIF of OCSD among patients with breast cancer in the training cohort**

| **Variable** | **N** | **1-y (%)** | **3-y (%)** | **5-y (%)** | **p** |
| --- | --- | --- | --- | --- | --- |
| **Total** | 3083 | 1.46 8.11 16.06 | | |  |
| **Age** |  | | | | <0.0001 |
| **Age>90** | 1175 | 0.77 | 4.37 | 9.26 |  |
| **80-90** | 1506 | 2.18 | 13.30 | 25.98 |  |
| **90+** | 402 | 7.23 | 29.46 | 49.11 |  |
| **Race** |  | | | | <0.0001 |
| **White** | 2623 | 1.47 | 8.24 | 16.42 |  |
| **Black** | 246 | 1.72 | 9.43 | 16.76 |  |
| **Others** | 214 | 1.14 | 5.96 | 12.20 |  |
| **Insurance** |  | | | | 0.0001 |
| **No** | 7 | 1.47 | 5.10 | 17.02 |  |
| **Yes** | 3006 | 1.46 | 8.05 | 15.91 |  |
| **Unknow** | 70 | 1.41 | 12.18 | 26.17 |  |
| **Marital status** |  | | | | <0.0001 |
| **No** | 1987 | 1.90 | 10.33 | 19.61 |  |
| **Yes** | 925 | 0.98 | 5.31 | 11.37 |  |
| **Unknow** | 171 | 0.92 | 8.84 | 18.58 |  |
| **Grade** |  | | | | 0.0019 |
| **1** | 824 | 1.23 | 7.15 | 14.80 |  |
| **2** | 1450 | 1.47 | 8.14 | 16.40 |  |
| **3** | 809 | 1.71 | 9.21 | 16.92 |  |
| **T** |  | | | |  |
| **0** | 0 | 0.00 | 0.00 | 0.00 | <0.0001 |
| **1** | 1640 | 0.97 | 6.10 | 13.54 |  |
| **2** | 1125 | 2.05 | 11.26 | 20.05 |  |
| **3** | 182 | 2.01 | 10.59 | 21.09 |  |
| **4** | 136 | 5.97 | 18.52 | 25.54 |  |
| **N** |  | | | | 0.919 |
| **0** | 2359 | 1.35 | 7.93 | 16.16 |  |
| **1** | 518 | 1.80 | 8.55 | 15.54 |  |
| **2** | 132 | 1.69 | 9.09 | 16.22 |  |
| **3** | 74 | 2.03 | 9.18 | 15.77 |  |
| **ER** |  | | | | 0.680 |
| **Negative** | 395 | 1.79 | 9.14 | 15.91 |  |
| **Positive** | 2688 | 1.41 | 7.96 | 16.08 |  |
| **PR** |  | | | | 0.069 |
| **Negative** | 785 | 1.79 | 9.01 | 16.74 |  |
| **Positive** | 2298 | 1.35 | 7.83 | 15.84 |  |
| **HER2** |  |  |  |  | 0.771 |
| **Negative** | 2781 | 1.44 | 8.04 | 16.16 |  |
| **Positive** | 302 | 1.64 | 8.73 | 15.10 |  |
| **Surgery** |  |  |  |  | <0.0001 |
| **No** | 300 | 6.07 | 24.54 | 35.49 |  |
| **Partial mastectomy** | 2270 | 1.13 | 6.92 | 14.56 |  |
| **Mastectomy** | 505 | 1.84 | 9.99 | 18.94 |  |
| **Other or unknow** | 8 | 2.56 | 10.87 | 26.76 |  |
| **Radiation** |  |  |  |  | <0.0001 |
| **No** | 2152 | 2.24 | 11.52 | 21.62 |  |
| **Yes** | 913 | 0.61 | 4.41 | 10.02 |  |
| **Chemotherapy** |  | | | | <0.0001 |
| **No** | 2820 | 1.60 | 8.80 | 17.51 |  |
| **Yes** | 263 | 0.71 | 4.44 | 8.19 |  |

CIF: Cumulative Incidences Function; OCSD: Other cause-specific death; T: tumor stage; N: nearby lymph node stage; ER: estrogen receptor; PR: progesterone receptor; HER2: growth factor receptor 2
